# Supplementary material for: Respiratory Auscultation Lab Using a Cardiopulmonary Auscultation Simulation Manikin
Source: MedEdPORTAL. 2021 Mar 2;17:11107. doi: 10.15766/mep_2374-8265.11107 (PMC7970645; doi:10.15766/mep_2374-8265.11107)
Supplement: Supplementary file 1 — Programming List.docxFacilitator Manual.docxStudent Manual.docxPostlab Discussion.docxStudent Feedback Form.docx [file mep_2374-8265.11107-s001.zip › A. Programming List.docx]

Please refer to the following list when programming the manikin. Lung sounds are listed in order of cases in the ‘Respiratory Exam and Lung Sounds Lab’ manual.

Group cases:

1. Normal vesicular breath sounds
2. Normal bronchial breath sounds
3. Rales
4. Wheezing
5. Rhonchi
6. Bronchial-vesicular breath sounds
7. Stridor
8. Pleural rub

Individual auscultation cases:

1. Left pleural friction rub

2. Egophony

3. Whispered pectoriloquy

4. Bronchophony
