# Supplementary material for: Respiratory Auscultation Lab Using a Cardiopulmonary Auscultation Simulation Manikin
Source: MedEdPORTAL. 2021 Mar 2;17:11107. doi: 10.15766/mep_2374-8265.11107 (PMC7970645; doi:10.15766/mep_2374-8265.11107)
Supplement: Supplementary file 1 — Programming List.docxFacilitator Manual.docxStudent Manual.docxPostlab Discussion.docxStudent Feedback Form.docx [file mep_2374-8265.11107-s001.zip › C. Student Manual.docx]

**Student Manual: Respiratory Exam and Lung Sounds Lab**

**Body Systems II: Cardiovascular, Respiratory, and Renal Systems**

**Session Title: Clinical Skills Lab: Respiratory Exam and Lung Sounds**

**Description**

● Coaching and practicing the skill of auscultating and interpreting physiologic and pathophysiologic lung sounds

● The session will begin with a lab and discussion on lung sounds with the opportunity to auscultate the lung sounds simulator.

**Readings**

Bates’ Guide to Physical Examination and History Taking, 11th edition:

pp 293-299 (Anatomy and physiology)

pp 305-315 (Techniques of examination)

pp 315-318 (Examination of the anterior chest)

pp 319 (Special techniques and recording the physical examination)

**Equipment**: Stethoscope

**Pre/Post session assignments:**

No pre- or post-session assignments

**Educational objectives:**

By the end of this program, learners will be able to:

1. Systematically auscultate the lungs for normal and abnormal breath sounds on a simulation manikin.
2. Identify and describe normal breath sounds.
3. Identify and describe abnormal breath sounds (crackles, wheezing, rhonchi, rales, stridor).
4. Interpret abnormal breath sounds in the context of a patient and/or disease.

**PHYSICAL EXAM SESSION FORMAT:**

Your students will have 90 minutes to learn and practice the technique of performing a lung exam.

**SESSION LOGISTICS OVERVIEW:**

The session is divided into 3 parts. During the first 50 minutes, 10 to 14 students participate in a facilitator-led large group discussion pertaining to 8 vignettes with their associated lung sounds and probing questions. The lung sounds for the first portion are played through the external speakers of the manikin. For the next 35 minutes, pairs of students break off for 5 minutes to run 1 additional vignette with the facilitator while directly auscultating the manikin. The remaining students practice the pulmonary physical exam while they await their turn to auscultate the manikin. Each pair analyzes the same vignette. The session ends with a 5 minute debrief.

**0-50 minutes: Lung Sounds Lab utilizing auscultation manikin**

**50-85 minutes: Practice cases utilizing auscultation manikin;**

● Each pair of students should take 5 minutes to examine the auscultation manikin in conjunction with the case and answer the probing questions.

**85-90 minutes: Debrief**

● Review the strengths and difficulties encountered by the students during the exam.

● Wrap up and final thoughts

The SAM II Auscultation Manikin is the basis for this lung sounds lab, but any auscultation manikin can be utilized. Each section is introduced with a small case and discussion questions that you should answer while examining each of the lung sounds. In order to improve clinical reasoning, you should predict which lung sound(s) are expected for each clinical scenario prior to listening to the sounds. Once the sound has been identified, a series of discussion questions follow.

**Case #1**

Case: Gwendolyn Adams, a healthy 32-year-old woman, has had a temperature of 101.3 with rhinorrhea and sore throat for the past 2 days. She has a scant non-productive cough. She is not short of breath. She did not take the influenza vaccination this year and is concerned about pneumonia. She comes to see you in the office and you auscultate her lungs and hear the following:

1. Based on the case what lung sounds do you expect to hear?
2. Describe what you are hearing:

**Case #1**

Case: Gwendolyn Adams, a healthy 32-year-old woman, has had a temperature of 101.3 with rhinorrhea and sore throat for the past 2 days. She has a scant non-productive cough. She is not short of breath and is very well appearing. She did not take the influenza vaccination this year and is concerned about pneumonia. She comes to see you in the office and you auscultate her lungs and hear the following:

Discussion questions as you are examining **Case #1:**

1. What are these sounds called?
2. Are these sounds normal?
3. How would you describe normal vesicular breath sounds in relation to the respiratory cycle?
4. Where in the airways do vesicular breath sounds originate?
5. Where are vesicular breath sounds auscultated?
6. What is Inspiration/Expiration (I/E) ratio? What is the I/E ratio in normal respiration?

**Case #2**

Case: You now move your stethoscope from Ms. Adam’s lateral lung fields medially to the second and third intercostal space and hear the following lung sounds:

1. Based on the case, what would you expect to hear?
2. Describe what you are hearing:

**Case #2**

Case: You now move your stethoscope from Ms. Adam’s lateral lung fields medially to the second and third intercostal space and hear the following lung sounds:

Discussion questions as you are examining **Case #2**:

1. What are these lung sounds called?
2. Where in the airways do bronchial breath sounds originate?
3. Are these breath sounds normal?
4. Where anatomically is bronchial breathing normal?
5. How do these sounds compare to the previous sound?

**Case #3**

Case: John Waters, a 67-year-old man with a history of systolic dysfunction, has been getting more dyspneic on walking 2 blocks, has some pedal edema and orthopnea. Auscultating his lungs, you hear the following sounds:

1. Based on the case what lung sounds do you expect to hear?
2. Describe what you are hearing:

**Case #3**

Case: John Waters, a 67-year-old man with a history of systolic dysfunction, has been getting more dyspneic on walking 2 blocks, has some pedal edema and orthopnea. Auscultating his lungs, you hear the following sounds:

Discussion questions as you are examining **Case #3**:

1. What are these lung sounds called?

2. Where in the respiratory cycle do rales occur?

3. What is the etiology of rales?

4. After starting treatment, you reassess the patient at the bedside. He is still tachypneic and retracting. How does his lung sounds differ from previous?

**Case #4**

Case: A 35-year-old female with a history of seasonal allergies presents to your office in April. She states she always gets short of breath in the springtime, which is associated with chest tightness. Last week she visited her friend’s house that has a cat and since she returned home her symptoms have worsened. Last year, her physician prescribed an inhaler, which seemed to have helped her symptoms.

1. Based on the case, what lung sounds do you expect to hear?
2. Describe what you are hearing:

**Case #4**

Case: A 35-year-old female with a history of seasonal allergies presents to your office in April. She states she always gets short of breath in the springtime, which is associated with chest tightness. Last week she visited her friend’s house that has a cat and since she returned home her symptoms have worsened. Last year, her physician prescribed an inhaler, which seemed to have helped her symptoms.

Discussion questions as you are examining **Case #4**:

1. What are these lung sounds called?
2. What is the etiology of wheezing?
3. Is this upper or lower airway pathology?
4. Where in the respiratory cycle does wheezing typically start?
5. Why does wheezing in asthma start in expiration before becoming continuous?
6. What are some conditions that can cause wheezing?
7. What is happening if you are seeing an asthmatic who is tachypneic and retracting but you do not hear any wheezing?

**Case #5**

Case: Emily McCarthy is a 76-year-old man with a 56-pack year history of smoking unfiltered Camel cigarettes. She states she has been having increased sputum production from baseline. She is complaining of shortness of breath and chest tightness. On listening to her lungs, you hear the following:

1. Based on the case what lung sounds do you expect to hear?
2. Describe what you are hearing:

**Case #5**

Case: Emily McCarthy is a 76-year-old woman with a 56-pack year history of smoking unfiltered Camel cigarettes. She states she has been having increased sputum production from baseline. She is complaining of shortness of breath and chest tightness. On listening to her lungs, you hear the following:

Discussion questions as you are examining **Case #5:**

1. What are these lung sounds called?

2. Where in the respiratory cycle are rhonchi heard?

3. What is the difference between rales and rhonchi?

4. How does this sound differ from wheezing?

5. Why do rhonchi and wheezes sound differently?

**Case #6**

Case: Richard Delacroix, a 65-year-old male with a history of chronic alcohol abuse, had a presumed viral upper respiratory infection one week ago. He now presents with the onset of a rigor, pleuritic chest pain on the left side and rust colored sputum. You listen to his lungs and hear the following:

1. Based on the case what lung sounds do you expect to hear?
2. Describe what you are hearing:

**Case #6**

Case: Richard Delacroix, a 65-year-old male with a history of chronic alcohol abuse, had a presumed viral upper respiratory infection one week ago. He now presents with the onset of a rigor, pleuritic chest pain on the left side and rust colored sputum. You listen to his lungs and hear the following:

Discussion questions as you are examining **Case #6:**

1. What is this type of respiration called?

2. Why does he have bronchial breathing?

3. What is the difference between vesicular and bronchial breathing?

4. How does the I:E ratio differ between normal vesicular breathing and bronchial breathing?

5. Where anatomically is bronchial breathing normal?

6. What are some causes of abnormal bronchial breathing?

**Case #7**

Case: Mr. Delacroix had a rough time with his pneumonia and was intubated for about 8 days. After discharge, he sees you 4 weeks later complaining of difficulty breathing. You listen to him and hear the following:

1. Based on the case what lung sounds do you expect to hear?
2. Describe what you are hearing while auscultating the patient’s trachea:

**Case #7**

Case: Mr. Delacroix had a rough time with his pneumonia and was intubated for about 8 days. After discharge, he sees you 4 weeks later complaining of difficulty breathing. You listen to him and hear the following:

Discussion questions as you are examining **Case #7:**

1. What is this sound called?

2. What does stridor indicate?

3. How does mild stridor compare to severe stridor in regards to the respiratory cycle?

4. What are some of the causes of stridor?

**Case #8**

Case: A 35-year-old female with a history of lupus presents with shortness of breath and chest pain. She has had a fever for several days associated with a non-productive cough. She states the chest pain is associated with inspiration and expiration and is very painful.

1. Based on the case what lung sounds do you expect to hear?
2. Describe what you are hearing while auscultating:

**Case #8**

Case: A 35-year-old female with a history of lupus presents with shortness of breath and chest pain. She has had a fever for several days associated with a non-productive cough. She states the chest pain is associated with inspiration and expiration and is very painful.

Discussion questions as you are examining **Case #8**:

1. What is this sound called?

2. What side of the stethoscope is used to best auscultate a pleural rub?

3. Where are pleural rubs best heard?

4. What do pleural rubs signify?

5. How do you differentiate a pleural friction rub from a pericardial friction rub?

**Individual auscultation manikin case**

Case: Richard Delacroix, a 65-year-old male with a history of chronic alcohol abuse, had a presumed viral upper respiratory infection one week ago. He now presents with the onset of a rigor, pleuritic chest pain on the left side and rust colored sputum. You listen to his lungs and hear the following:

Based on the case, what lung sounds and findings do you expect to hear?

**Sound #1**

1. Describe what you are hearing:
2. What does this sound indicate?

**Sound #2**

1. What is this sound called?
2. What is egophony?
3. How do you elicit egophony?
4. What does this sound indicate?

**Sound #3**

1. What is this sound called?
2. What is whispered pectoriloquy?
3. How do you elicit whispered pectoriloquy?
4. What does whispered pectoriloquy indicate?

**Sound #4**

1. What is this sound called?
2. What is bronchophony?
3. How do you elicit bronchophony?
4. Which words in the English language are better suited to elicit bronchophony and why?
5. What does bronchophony indicate?

**DEBRIEF**

● What was the most difficult part of this laboratory session?

● What do you feel like went particularly well, and what would you do to change the session in the future?

● What are some challenges that you encountered while auscultating lung sounds on the manikin? Did you feel comfortable differentiating between the lung sounds?
