# Supplementary material for: Respiratory Auscultation Lab Using a Cardiopulmonary Auscultation Simulation Manikin
Source: MedEdPORTAL. 2021 Mar 2;17:11107. doi: 10.15766/mep_2374-8265.11107 (PMC7970645; doi:10.15766/mep_2374-8265.11107)
Supplement: Supplementary file 1 — Programming List.docxFacilitator Manual.docxStudent Manual.docxPostlab Discussion.docxStudent Feedback Form.docx [file mep_2374-8265.11107-s001.zip › D. Postlab Discussion.docx]

**Post-Respiratory Sounds Lab Discussion**

**Case #1**

**Normal Vesicular Breath Sounds**

Case: Gwendolyn Adams, a healthy 32-year-old woman, has had a temperature of 101.3 with rhinorrhea and sore throat for the past 2 days. She has a scant non-productive cough. She is not short of breath. She did not take the influenza vaccination this year and is concerned about pneumonia. She comes to see you in the office and you auscultate her lungs and hear the following:

Based on the case what lung sounds do you expect to hear?

1. The patient has signs and symptoms of an upper respiratory infection. One expects to hear normal breath sounds.

What are you hearing?

1. A hollow blowing sound that is even and low pitched. It is present during inspiration and expiration.

Discussion questions as you are examining the normal vesicular lung sounds:

1. What are these sounds called?
   1. Vesicular breath sounds
2. Are these sounds normal?
   1. Yes
3. How would you describe normal vesicular breath sounds in relation to the respiratory cycle?
   1. Vesicular breath sounds are greatest in inspiration and diminish in end expiration.
4. Where in the airways do vesicular breath sounds originate?
   1. Vesicular breath sounds occur from the normal movement of air through the bronchioles.
5. Where are vesicular breath sounds auscultated?
   1. Vesicular breath sounds are heard over the thorax and arise in the periphery. Vesicular breath sounds are heard directly overlying lung tissue.
6. What is Inspiration/Expiration (I/E) ratio? What is the I/E ratio in normal respiration?
   1. Inspiration/Expiration ratio is the ratio of duration of inspiration to the duration of expiration. In an adult, the normal I:E ratio is 3:1. In vesicular breathing, there is no pause between inspiration and expiration.

**Case #2**

**Normal Bronchial Breath Sounds**

Case: You now move your stethoscope from Ms. Adam’s lateral lung fields medially to the 2^nd^ and 3^rd^ intercostal space and hear the following lung sounds:

Based on the case, what would you expect to hear?

1. Bronchial breath sounds are best heard over the 2^nd^ and 3^rd^ intercostal space.

Describe what you are hearing:

1. Loud, high-pitched tubular and hollow sounds

Discussion questions as you are examining bronchial breath sounds:

1. What are these lung sounds called?
   1. Bronchial breath sounds
2. Where in the airways do bronchial breath sounds originate?
   1. Bronchial breath sounds are heard as air passes through the tracheobronchial tree.
3. Are these breath sounds normal?
   1. Depends on the location where they are heard. In this case, at the manubrium they are considered normal.
4. Where anatomically is bronchial breathing normal?
   1. The trachea, right sternoclavicular joints, and posterior right interscapular space are the only locations where tracheobronchial trees sit close to the chest wall without surrounding lung tissue.
5. How do bronchial breath sounds compare to the previous vesicular breath sounds?
   1. Bronchial sounds are more tubular and hollow sounding than vesicular sounds. They are typically louder and higher in pitch than vesicular breath sounds.

**Case #3**

**Rales**

Case: John Waters, a 67-year-old man with a history of systolic dysfunction, has been getting more dyspneic on walking two blocks, has some pedal edema and orthopnea. Auscultating his lungs, you hear the following sounds:

Based on the case what lung sounds do you expect to hear?

1. This patient has signs and symptoms of CHF. You would expect to hear crackles/rales.

Describe what you are hearing:

1. Brief, discontinuous, popping lung sounds that are high-pitched. Fine crackles are also similar to the sound of wood burning in a fireplace, or hook and loop fasteners being pulled apart or cellophane being crumpled.

Discussion questions as you are examining rales:

1. What are these lung sounds called?
   1. Crackles or rales
2. Where in the respiratory cycle do rales occur?
   1. Rales occur in inspiration and expiration but are discontinuous.
3. What is the etiology of rales?
   1. It is believed due to the sudden opening of collapsed distal airways during inspiration. Rales are not always pathological; they can occur from postural atelectasis (a condition where the [alveoli](http://en.wikipedia.org/wiki/Alveoli) are deflated) in the lungs but will clear with coughing.
4. After starting treatment, you reassess the patient at the bedside. He is still tachypneic and retracting. How does his lung sounds differ from previous?
   1. The patient now has coarse crackles. Coarse crackles are discontinuous, brief, popping lung sounds. Compared to fine crackles they are louder, lower in pitch and last longer. They have also been described as a bubbling sound. You can simulate this sound by rolling strands of hair between your fingers near your ear.

**Case #4**

**Wheezing**

Case: A 35-year-old female with a history of seasonal allergies presents to your office in April. She states she always gets short of breath in the springtime, which is associated with chest tightness. Last week, she visited her friend’s house that has a cat, and since she returned home her symptoms have worsened. Last year, her physician prescribed an inhaler, which seemed to have helped her symptoms.

Based on the case, what lung sounds do you expect to hear?

1. The patient has signs and symptoms of reactive airway disease or asthma. One would expect to hear wheezing on exam.

Describe what you are hearing:

1. Continuous high-pitched sounds, most pronounced at end expiration

Discussion questions as you are examining wheezing:

1. What are these lung sounds called?
   1. Wheezes
2. What is the etiology of wheezing?
   1. Wheezing is caused by turbulent flow of air through an airway that is obstructed from swelling or secretions. During inspiration, the elasticity of the airways will cause them to dilate which will allow air to flow around the obstruction. As the airways contract during exhalation, airflow will increase causing the high-pitched sounds associated with wheezing.
3. Is this upper or lower airway pathology?
   1. Wheezing occurs in the lower airways.
4. Where in the respiratory cycle does wheezing typically start?
   1. In expiration
5. Why does wheezing in asthma start in expiration before becoming continuous?
   1. Initially, the elasticity of the airways causes dilatation of the airways during inspiration. As the obstruction worsens the elasticity of the airways is lost and air can no longer flow around the obstruction in inspiration.
6. What are some conditions that can cause wheezing?
   1. Reactive airway disease, asthma, COPD, foreign body ingestion especially if unilateral
7. What is happening if you are seeing an asthmatic who is tachypneic and retracting but you do not hear any wheezing?
   1. This patient is in severe respiratory distress.

**Case #5**

**Rhonchi**

Case: Emily McCarthy is a 76-year-old woman with a 56-pack year history of smoking unfiltered Camel cigarettes. She states she has been having increased sputum production from baseline. She is complaining of shortness of breath and chest tightness. On listening to her lungs, you hear the following:

Based on the case what lung sounds do you expect to hear?

1. Rhonchi and possibly wheezing

Describe what you are hearing:

- 1. Continuous, both inspiratory and expiratory, low-pitched adventitious lung sounds. They are described as snoring or gurgling.

Discussion questions as you are examining rhonchi/low-pitched wheezes:

1. What are these lung sounds called?
   1. Rhonchi
2. Where in the respiratory cycle are rhonchi heard?
   1. They are heard in both inspiration and expiration because they are caused by secretions.
3. What is the difference between rales and rhonchi?
   1. Rales are discontinuous sounds and rhonchi are continuous. Rhonchi are a low-pitched wheeze and therefore continuous.
4. How do rhonchi differ from wheezing?
   1. Wheezes are high-pitched expiratory sounds while rhonchi are low-pitched. Rhonchi are made up of low-pitched wheezes.
5. Why do rhonchi and wheezes sound differently?
   1. Rhonchi generally occur in larger airways and are typically caused by obstruction from secretions. This affects both the inspiratory and expiratory cycle.

**Case #6**

**Bronchial-vesicular breathing**

Case: Richard Delacroix, a 65-year-old male with a history of chronic alcohol abuse, had a presumed viral upper respiratory infection one week ago. He now presents with the onset of a rigor, pleuritic chest pain on the left side and rust colored sputum. You listen to his lungs and hear the following:

Based on the case what lung sounds do you expect to hear?

1. The patient has signs and symptoms of pneumonia. Correct answers include rales, pleural rub, bronchial breath sounds in the periphery.

Describe what you are hearing:

1. Hollow, tubular sounds that are lower pitched in the periphery overlaying the lung fields in the periphery

Discussion questions as you are examining abnormal bronchial breath sounds

1. What is this type of respiration called?
   1. Bronchial breathing-the presence of bronchial breath sounds in areas where vesicular sounds should be auscultated.
2. Why does he have bronchial breathing?
   1. The alveoli are filled with fluid and allow the transmission of the central airways to the periphery. Fremitus is also increased by the same mechanism.
3. What is the difference between vesicular and bronchial breathing?
   1. Vesicular breath sounds inspiration is longer than expiration. It is felt the inspiratory sounds originate from the peripheral airways and the expiratory component from the more proximal airways. With bronchial breath sounds expiration is longer than inspiration and these sounds originate from the more proximal airways. They are loud and harsher than vesicular sounds.
4. How does the I:E ratio differ between normal vesicular breathing and bronchial breathing?
   1. Bronchial sounds expiration is longer than inspiration. The bronchial I:E ratio generally is 1:3, which is abnormal.
5. Where anatomically is bronchial breathing normal?
   1. Over the trachea, right sternoclavicular joints, and posterior right interscapular space
6. What are some causes of abnormal bronchial breathing?
   1. Consolidation, cavitation, pneumothorax, massive pleural effusion with complete atelectasis, mass superimposed between the chest wall and large airways

**Case #7**

**Stridor**

Case: Mr. Delacroix had a rough time with his pneumonia and was intubated for about 8 days. After discharge he sees you 4 weeks later complaining of difficulty breathing. You listen to him and hear the following:

Based on the case what lung sounds do you expect to hear?

1. The patient is at risk for tracheal stenosis from the prolonged intubation. You would expect to hear stridor.

Describe what you are hearing while auscultating the patient’s trachea:

1. It is a musical and involves both inspiration and expiration. This sound is loudest when listening over the neck.

Discussion questions as you are examining stridor:

1. What is this sound called?
   1. Stridor
2. What does stridor indicate?
   1. Upper airway obstruction
3. How does mild stridor compare to severe stridor in regards to the respiratory cycle?
   1. Mild stridor is confined to inspiration. As stridor worsens it may be heard throughout the entire respiratory cycle. If stridor is severe it may be audible without a stethoscope.
4. What are some of the causes of stridor?
   1. Epiglottitis, croup, foreign body, vocal cord paralysis, tumor. It is also seen in up to 35% of extubated patients.

**Case #8**

**Pleural Rub**

Case: A 35-year-old female with a history of lupus presents with shortness of breath and chest pain. She has had a fever for several days associated with a non-productive cough. She states the chest pain is associated with inspiration and expiration is very painful.

Based on the case what lung sounds do you expect to hear?

1. The patient is describing pleuritic chest pain. Her history of lupus puts her at risk for inflammation of the pleural lining, which would give rise to a pleural rub.

Describe what you are hearing while auscultating:

1. A high frequency grating, scratchy sound

Discussion questions as you are examining a pleural rub:

1. What is this sound called?
   1. A pleural rub
2. What side of the stethoscope is used to best auscultate a pleural rub?
   1. The diaphragm because it is a high-pitched sound
3. Where are pleural rubs best heard?
   1. Over the anterolateral chest wall. One sound is heard on inspiration and one on expiration. Although the sounds may be discrete, sometimes they are so numerous that they can merge to form a continuous sound as well. Pleural rubs may be transient.
4. What do pleural rubs signify?
   1. They are caused by inflamed pleural lining rubbing together. Common causes include viral infections such as coxsackie, influenza, inflammatory processes such as lupus, tuberculosis, and lung cancer and have been attributed to pulmonary embolus.
5. How do you differentiate a pleural friction rub from a pericardial friction rub?
   1. Pleural friction rubs disappear with breath-holding. Pleural rubs are louder in expiration while pericardial rubs are louder during inspiration and do not disappear with breath holding. Pericardial rubs are best heard over the left sternal border.

**Individual auscultation manikin case**

Case: Richard Delacroix, a 65-year-old male with a history of chronic alcohol abuse, had a presumed viral upper respiratory infection one week ago. He now presents with the onset of a rigor, pleuritic chest pain on the left side and rust colored sputum. You listen to his lungs and hear the following:

Based on the case what lung sounds and findings do you expect to hear?

1. Correct answers include-rales, bronchial breath sounds, pleural rub, egophony, whispered pectoriloquy, and bronchophony.

**Sound #1**

**Pleural Rub Left**

Describe what you are hearing:

1. The right side of the lung has normal vesicular breath sounds. The left side has a high-pitched scratchy sound that disappears with breath holding.

What does this sound indicate?

1. The patient has a pleural friction rub, indicating inflammation of the pleura, most likely from an underlying consolidation. Stress to the students the importance of auscultating bilaterally since the right side is normal in this case.

**Sound #2**

**Egophony**

Describe what you are hearing:

1. There is a voiced sound with a nasal quality, akin to a goat's bleating. Egophony has higher intensity over abnormal lung areas. The sound is heard as an "A" (aaay) indicating underlying consolidation.

Discussion questions as you examine egophony:

1. What is this sound called?
   1. Egophony
2. What is egophony?
   1. Egophony is the E to A change heard over an area of consolidation.
3. How do you elicit egophony?
   1. Ask the patient to say "Eeee" several times and auscultate the chest walls. Over healthy lung areas, the sound is understandable as an "E".
4. What does this sound indicate?
   1. The patient has an underlying consolidation.

**Sound #3**

**Whispered Pectoriloquy**

Describe what you are hearing:

1. The whispered word is intensified. The transmission of spoken words is increased, and the spoken word is clearly heard. The numbers 1-2-3 are clearly heard. There is intensification of the patient’s whispered voice.
2. What is this sound called?
   1. Whispered pectoriloquy
3. What is whispered pectoriloquy?
   1. Whispered pectoriloquy is intensification of a patient’s whispered voice over an area of consolidation. Transmission of spoken words is increased and the spoken word is clearly heard.
4. How do you elicit whispered pectoriloquy?
   1. Have the patient whisper one-two-three while auscultating all lung fields.
5. What does whispered pectoriloquy indicate?
   1. Whispered pectoriloquy indicates underlying consolidation.

**Sound #4**

**Bronchophony**

Describe what you are hearing:

1. The word “ninety-nine” is clearly heard over the lateral lung fields. In the peripheral lung fields the voice should become softer and less distinct.
2. What is this sound called?
   1. Bronchophony
3. What is bronchophony?
   1. Bronchophony is intensification of a patient’s voice over an area of consolidation. Transmission of spoken words is increased and the spoken word ninety-nine is clearly heard.
4. How do you elicit bronchophony?
   1. Have the patient say, “ninety-nine” while auscultating all lung fields.
5. Which words in the English language are better suited to elicit bronchophony and why?
   1. Toy boat, blue balloon. The word “ninety-nine” is a direct translation of the German word, “Neun und Neunzig” which would cause maximum vibration of the chest. In the English language the phrases, “toy boat or blue balloon” would bring a higher yield.
6. What does bronchophony indicate?
   1. Bronchophony indicates underlying consolidation.
