# Supplementary material for: Respiratory Auscultation Lab Using a Cardiopulmonary Auscultation Simulation Manikin
Source: MedEdPORTAL. 2021 Mar 2;17:11107. doi: 10.15766/mep_2374-8265.11107 (PMC7970645; doi:10.15766/mep_2374-8265.11107)
Supplement: Supplementary file 1 — Programming List.docxFacilitator Manual.docxStudent Manual.docxPostlab Discussion.docxStudent Feedback Form.docx [file mep_2374-8265.11107-s001.zip › E. Student Feedback Form.docx]

**Lung Sounds Lab and Respiratory Exam (Clinical Skills Lab)**

**Lung Sounds Lab and Respiratory Exam (Clinical Skills Lab)**
Please rate the effectiveness of this activity in facilitating your learning. Include in your consideration the assigned pre-readings, your level of engagement, and the clarity of the learning objectives. Please select a rating (radio button) below. A space for written comments is also provided. Written comments are valuable but are not required - the written comment box is optional.

- Extremely effective **(5)**
- Very effective **(4)**
- Average **(3)**
- Somewhat effective **(2)**
- Not at all effective **(1)**

Comment on **Lung Sounds Lab and Respiratory Exam (Clinical Skills Lab)**

________________________________________________________________

________________________________________________________________

________________________________________________________________

________________________________________________________________

________________________________________________________________
