# Supplementary material for: Respiratory Auscultation Lab Using a Cardiopulmonary Auscultation Simulation Manikin
Source: MedEdPORTAL. 2021 Mar 2;17:11107. doi: 10.15766/mep_2374-8265.11107 (PMC7970645; doi:10.15766/mep_2374-8265.11107)
Supplement: Supplementary file 1 — Programming List.docxFacilitator Manual.docxStudent Manual.docxPostlab Discussion.docxStudent Feedback Form.docx [file mep_2374-8265.11107-s001.zip › _Educational_Summary_Report_11107.pdf]

MedEdPORTAL® The AAMC Journal of Teaching and Learning Resources

Original Publication

Open Access

Respiratory Auscultation Lab Using a Cardiopulmonary Auscultation Simulation Manikin

Jennifer Kaminsky, MD*, Riccardo Bianchi, PhD, Shirley Eisner, PhD, Robin Ovitsh, MD, Ana Maria Lopez, Leanna Smith, Nawar Talukder, MD, Antonia Quinn, DO

*Corresponding author: JKaminsky1@northwell.edu

Abstract

Introduction: Mastery of respiratory auscultation skills is fundamental for clinicians to develop. We created a case-based educational session utilizing a high-fidelity simulator to teach lung sound auscultation to medical students at our institution. We employed a hypothesis-driven approach and deliberate practice to enhance students' learning experience and retention of acquired skills. **Methods:** We developed the session to teach second-year medical students how to discriminate between normal and pathological respiratory sounds within the context of clinical vignettes. Faculty facilitators, in conjunction with near-peer educators, made use of a high-fidelity auscultation manikin to guide students through case-based problem sets. Students were given the opportunity to auscultate the manikin while being observed and receiving feedback from the faculty. **Results:** We introduced the manikin in 2016, with a total of 759 second-year medical students from four class years having participated in the session since then. Students evaluated the session through an end-of-the-week and end-of-unit survey. The survey showed an overall improvement in learner satisfaction over previous years. Survey results and feedback were used to make adjustments to the session. **Discussion:** Our respiratory auscultation session was well received overall. Proper faculty development is crucial for implementing the session. Because of the focus on deliberate practice, adequate time must be allotted to hold the session. This program is reproducible with similar high-fidelity simulators.

Keywords
Respiratory Auscultation, Deliberate Practice, Hypothesis-Driven Physical Examination (HDPE), Simulation, High Fidelity, Lung Sounds, Case-Based Learning, Laboratory Education, Pulmonary Medicine, Clinical/Procedural Skills Training

Educational Objectives

By the end of this session, learners will be able to:

1. Systematically auscultate the lungs for normal and abnormal breath sounds on a simulation manikin.
2. Identify and describe normal breath sounds.
3. Identify and describe abnormal breath sounds (crackles, wheezing, rhonchi, rales, stridor).
4. Interpret abnormal breath sounds in the context of a patient and/or disease.

Citation:
Kaminsky J, Bianchi R, Eisner S, et al. Respiratory auscultation lab using a cardiopulmonary auscultation simulation manikin. *MedEdPORTAL*. 2021;17:11107.
https://doi.org/10.15766/mep_2374-8265.11107

Introduction

Respiratory auscultation and the ability to differentiate between normal and pathologic lung sounds are fundamental clinical skills for learners across various fields of medicine. However, medical students and nonpulmonologist physicians often struggle with differentiating basic lung sounds upon auscultation.[1] The medical curriculum at SUNY Downstate Health Sciences University includes traditional lectures, problem-based learning, and team-based learning. While students are presented with the material across these various formats, opportunities to directly apply their knowledge of respiratory anatomy and physiology in preclinical settings may still be rather limited.[2] High-fidelity simulation within a clinical context increases understanding of lung sounds physiology, auscultation skills, engagement of learners, and retention of acquired skills.[3] We created an educational session using a high-fidelity cardiopulmonary simulator (SAM II cardiac manikin, Cardionics) to teach respiratory auscultation to students at our institution. However, any simulator producing high-fidelity respiratory sounds could have been used for this session.

Copyright © 2021 Kaminsky et al. This is an open-access publication distributed under the terms of the Creative Commons Attribution license.

Our program was grounded in deliberate practice as the primary educational framework. Our lung sounds session was modeled after our heart sounds session, previously published in *MedEdPORTAL*,[4] and these modules can be used as compendium resources. Deliberate practice provides novice learners with opportunities to hone fundamental skills through intentional, purposeful practice and repetition via guided instruction and feedback throughout the learning process.[5,6] Guided feedback and sufficient opportunities for repetitive practice and skill refinement often lead to significant improvement in performance.[7] Deliberate practice has been applied to both procedural and cognitive skills.[8] For example, cardiac auscultation has been previously taught using a deliberate practice framework.[7] We premised that the same framework could be applied to teaching the auscultation of lung sounds. Deliberate practice is especially important in the initial stages of learning a skill set (e.g., auscultation or other physical examination maneuvers).[9]

We replaced a web-based program with a high-fidelity auscultation manikin because the quality of lung sounds on the free, web-based program was often poor. Students and facilitators also noted that pop-up advertisements on the online program were distracting and that the online program was not always intuitive and convenient to navigate. Additionally, our revised educational session provided learners with hands-on practice time and opportunities to apply and translate their knowledge to a physical model, which was not possible with the prerecorded sounds. Specifically, the SAM II simulator accurately reflected human surface anatomy, which allowed learners to auscultate at the anatomically correct position for each lung sound.

This educational session employed a hypothesis-driven approach, which encouraged students to anticipate physical exam findings for a given clinical scenario.[10] This educational framework has been shown to increase learner retention and engagement by demonstrating clinical relevance of normal and pathologic findings. A hypothesis-driven approach, along with deliberate practice, aids in fostering clinical reasoning skills and provides learners with an opportunity to develop differential diagnoses in accordance with their findings.[4,11] In this program, students worked through a series of clinical vignettes and were prompted to anticipate normal and/or abnormal sounds for each case. Since the session was geared towards novice learners, the vignettes were level-set so that the anticipated sounds were readily deduced.

High-fidelity simulation has previously been employed to teach lung sounds. Bintley, Bell, and Ashworth divided students into groups of 10 to auscultate a simulation manikin.[2] In contrast, we had students work in pairs to practice correct stethoscope placement and auscultation skills on the manikin, which allowed maximal facilitator interaction and focused feedback.

We searched *MedEdPORTAL* for published curricula on the teaching of lung sounds to medical students and found two related curricula.[12,13] However, both those sessions involved the use of prerecorded normal and pathologic lung sounds to facilitate students' learning. One of the sessions utilized supplemental graphic aids (animation, graphic displays of acoustic signals) in addition to the recorded sounds.[13] Neither focused on high-fidelity simulation to teach sounds in real time.

Here, we describe our design and implementation of the lung sounds auscultation teaching session and the learner evaluation of this innovative program.

Methods

Target Audience

This session was created for second-year preclinical medical students. Our institution taught the session in association with the pulmonary block of the preclinical segment of the curriculum. However, it could function as a stand-alone session by teaching the respiratory exam and the associated lung sounds as isolated skills. While intended for medical students, this session could be equally useful in other health education settings. Physician assistant and nursing students or resident physicians could benefit from this session with appropriate level-setting for the learners.

Prerequisites

- Introduction and understanding of basic lung physiology and pathophysiology.
- Understanding of the surface anatomy and pertinent respiratory auscultation areas.

Preparation

In advance of the session, we entered the required lung sounds in sequence into the simulator manikin (Appendix A). We started with physiologic lung sounds to lay the groundwork for the study of pathologic sounds.

Each session was facilitated by a faculty member—at our institution, we used resident and attending physicians from various disciplines including pulmonology, internal medicine, emergency medicine, and pediatrics. We relied on physician

Copyright © 2021 Kaminsky et al. This is an open-access publication distributed under the terms of the Creative Commons Attribution license.

facilitators, but any advanced practitioner, such as a physician assistant or nurse practitioner, with appropriate background in pulmonary auscultation can lead the sessions.

Each faculty was accompanied by one near-peer educator (NPE). NPEs were senior medical students who supported faculty in executing the sessions. Using NPEs to help teach has been shown to aid learners in the acquisition and retention of physical diagnosis skills.[14] NPEs' recent experience with the same session and curriculum rendered them extremely helpful in cofacilitating and fielding questions from students, though faculty could carry out the sessions alone as well. Each faculty-NPE pair worked with a group of 14 students and one auscultation manikin.

We provided faculty and NPEs with a facilitator manual (Appendix B). Facilitators underwent approximately 1 hour of faculty development to receive general information about the students' level of preparation, to familiarize them with the session learning objectives and format, and to train them in the technological aspects of the simulator, such as advancing through the sounds. Prior to the session, facilitators familiarized themselves with the clinical vignettes and corresponding lung sounds. They also went over a list of probing questions to aid in their facilitation and challenge learners. The rest of the development session involved practicing auscultation on the manikin and progressing through the sounds on the simulator's laptop. There were no additional faculty development materials aside from the facilitator manual.

Prior to the lab, the Associate Dean for Clinical Competencies gave students an interactive lecture in which she demonstrated normal and pathological lung sounds utilizing the same manikin slated for use in the lab. Our institution used *Bates' Guide to Physical Examination and History Taking*[15] as the required physical examination textbook; however, any physical examination textbook would provide adequate prereading for the session.

Session Logistics

We utilized the SAM II auscultation manikin for this educational session—this model consisted of a portable stand-alone torso equipped with internal speakers (Figure). SAM II sat upright on a base and plugged into an electrical outlet. The manikin connected to a laptop via USB cable. While our institution relied on SAM II, this session could be adapted to any auscultation simulator, such as Harvey (Laerdal Medical)[16] or Ventriloscope (Lecat's Ventriloscope).

We scheduled the activity during a physical diagnosis session focused on mastering normal and pathological lung sounds, to be completed in small groups. We utilized a small-group setting because it afforded students the opportunity to auscultate the manikin themselves.

The lab session was scheduled for 1.5 hours, allowing for respiratory physical exam practice for the students with their clinical skills partners while giving them additional opportunity to solicit feedback from faculty on the exam. This publication includes materials for the programming and presentation of cases used for the lung sounds lab.

The program ran over 2 days. The sessions ran two times per day for an hour and a half each. We had four manikins available. The class of 210 students was divided into quarters and then four rooms, ending up with approximately 14 students per room, or seven pairs.

During the course of the lab, students were prompted to identify and interpret simulated lung sounds. We opted to alternate between normal and pathologic sounds to build confidence in discerning between the two. We provided vignettes and probing questions about each case to allow students to work through auscultatory findings they might expect, utilizing a hypothesis-driven approach.

Large-group case discussion (50 minutes): The basis of the lab was a case-based, hypothesis-driven approach to teaching lung auscultation. Facilitators presented students with eight clinical scenarios, each consisting of a short vignette, similar to a question stem (Appendix C). Facilitators had access to the scenarios, as well as corresponding probing questions and their correct interpretations (highlighted in red in their manuals). After listening to each vignette but before auscultating the manikin, students discussed which lung sounds they anticipated hearing. The facilitator played the sounds through the external speakers, allowing each student to listen before working through a series of questions. The probing questions related to anatomic landmarks for optimal auscultation sites, associated diagnoses, and pathophysiologic underpinnings of certain sounds. The SAM II program also provided a visual on a projected video screen showing the surface location of each auscultation point during the lab. One week after the session, students were given the answers to the probing questions (in Appendix D, answers highlighted in red).

Direct auscultation of manikin (35 minutes): The lung auscultation manikin had internal speakers, which allowed students to directly auscultate the simulator using their own stethoscopes. A pair of students directly auscultated the manikin for 5 minutes

Copyright © 2021 Kaminsky et al. This is an open-access publication distributed under the terms of the Creative Commons Attribution license.

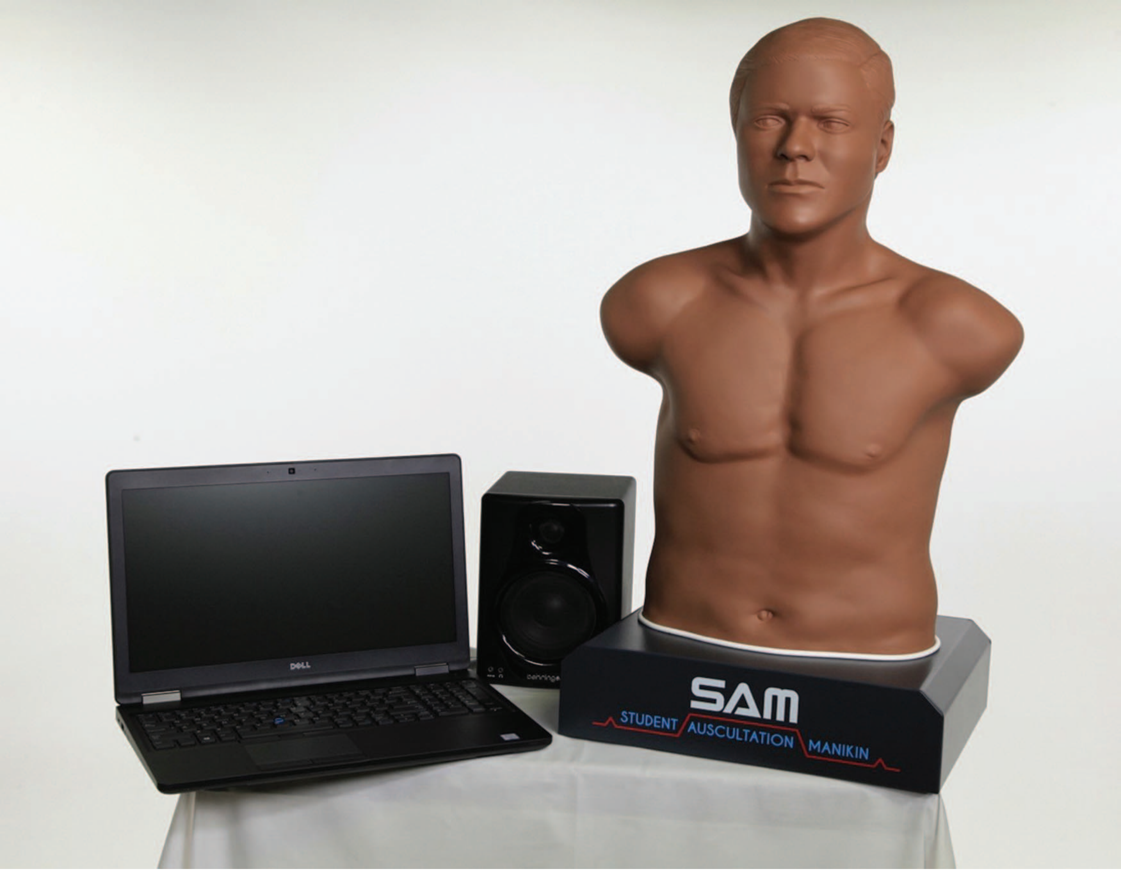

Figure. Image of SAM II manikin.

and analyzed lung sounds to evaluate an additional patient vignette. We felt that this was enough time as the case introduced bronchophony, egophony, and whispered pectoriloquy, which highlighted the same underlying pathology. The remaining students in the lab practiced the physical examination on each other while waiting to use the simulator. Individual institutions can adjust the duration of the session to suit their specific time constraints.

Debrief (5 minutes): We reserved 5 minutes for a debrief to discuss strengths and challenges encountered by students during the session.

With each class, this session was evaluated twice via a 5-point Likert-scale feedback form. This survey instrument was the standard utilized by all courses in the medical school and therefore was not specific to this session. The survey was completed once at the end of the week by the Course/Faculty Assessment (CFA) team and once at the end of the block by the entire class through open-box comments (Appendix E). The CFA consisted of a core group of 20 randomly selected students who were tasked with evaluating lectures, laboratories, and small-group sessions, in addition to the instructors who led them. Their evaluation occurred at the end of the week immediately following the session. The collated feedback produced by this group was then presented to clinical skills leadership. To assess whether the learning objectives had been met, an end-of-unit summative examination included a sampling of questions related to lung sounds.

Results

Since the initial debut of the manikin in 2016, a total of 759 students from four class years have been trained using the SAM II simulator. From 2016 to 2018, the class size averaged 183 students per year. In 2019, there was an increase in class size to 210, giving us a total of 759 students.

Since replacing the web-based free program with the auscultation manikin, student ratings of each iteration have continued to show improvement. In 2014 and 2015, prior to

Copyright © 2021 Kaminsky et al. This is an open-access publication distributed under the terms of the Creative Commons Attribution license.

the introduction of the manikin, 48% and 58% of the students, respectively, rated the sessions average or not at all effective. In 2016, 42% of students rated the session as average, while 25% rated the session as very effective or extremely effective. Through subsequent years, student ratings have improved, with 79% of students rating the sessions as either very effective or extremely effective by 2018. In 2019, student ratings of very or extremely effective dropped to 68%, while the remaining 32% rated the session as average or below. Even though the ratings decreased in 2019, they were still markedly higher than at the inception of the program and before the introduction of the manikin.

Several themes became apparent from student narrative feedback. Prior to the introduction of the manikin, students commented how difficult it was to distinguish lung sounds when not directed to the surface anatomy. After 2016, comments highlighted the benefit of the manikin for delineating the location of lung sounds. Furthermore, students consistently felt that the session helped them understand the difference between abnormal and normal lung sounds. Prior to the introduction of the manikin, students had stated that the static in the commercial product limited the ability of the session to achieve its goals. Once the manikin had been introduced, positive feedback reiterated that it was crucial in helping the students distinguish between different lung sounds and their corresponding location. Constructive comments mostly suggested that session times be lengthened in order to provide greater opportunity for repetition. Pre- and postintroduction of the manikin, students rated the session very favorably when they had received strong feedback from the facilitator. In 2019, several students felt some faculty were not as adept at using the technology, making them perceive that the faculty were "disconnected from the laboratory session." Below are some of the comments we received, arranged by themes:

- Surface anatomy (from 2015, prior to introduction of the manikin):
  - "It is really difficult to interpret sounds… especially if we don't know where on the body we are listening. In a real patient we don't put our stethoscope on them with our eyes closed."
- Sound quality (comments pooled from all iterations pre- and postmanikin):
  - "3-5 second clips of static filled sounds are not helpful" (2014).
  - "SAM II helps simulate real patient sounds."
- Content and learning objectives (comments pooled from all iterations pre- and postmanikin):
  - "Great way to finally put together different sounds with different clinical situations."
  - "This was a great session and really helped me to learn the expected findings with different forms of respiratory disease. I feel very confident about this subject matter after this session."
  - "Listening to the lung sounds helps provide repetition needed for distinguishing between them."
  - "It was extremely clarifying to go through the cases and practice on the manikin."
- Facilitation (comments pooled from all iterations pre- and postmanikin):
  - "[Facilitator name] was a great facilitator and made learning the information enjoyable. She was thorough and succinct with her explanations, which allowed us to digest the information."
  - "All credit to Dr. [facilitator name]. He helped us connect the sounds to the lung pathologies."
  - "Facilitator did not know how to use the SAM II and hindered the session as we were not able to put all the sounds to the cases. In the future always pair faculty with a senior student who knows what to do with the model."
- Duration of session (comments postintroduction of the manikin):
  - "A slightly extended session, say 30 minutes longer would have allowed coverage of all sounds. My group was a bit rushed."
  - "I would appreciate it if we had more time in general for this unit to practice with SAM II."
  - "We did not have time to listen to SAM II or practice the exam since we spent the entire time on cases."

Note that although student comments refer to the SAM II simulator, any simulator can be used for this session.

Discussion

To address a need for teaching lung sounds auscultation, we developed a session utilizing a cardiopulmonary auscultation manikin and employed a deliberate practice framework and hypothesis-driven approach. Overall, our session was very well received. Strengths included types of lung sounds and pulmonary pathologies covered, quality of cases presented, and probing questions. The end-of-week Likert-rating data, in conjunction with student feedback, suggested that this modality of teaching had a positive impact on the student learning experience.

Copyright © 2021 Kaminsky et al. This is an open-access publication distributed under the terms of the Creative Commons Attribution license.

The reviews of the session can be divided between design and delivery. Aspects of design that were well received included the session being case based and facilitated with concrete feedback, as well as the content being appropriate for the level of learner. The students felt able to distinguish normal and abnormal lung sounds as per the learning objectives of the session. Based on course evaluations, students perceived an increase in learning of lung sounds (level I of the Kirkpatrick model of evaluation).[17] Prior to the initiation of the manikin, comments centered on the strength of the cases and the hypothesis-driven approach. The students valued the personalized feedback from the facilitators on distinguishing lung sounds and relating them to the specific cases. From student evaluations of the session, it is apparent that coaching, as well as opportunities for deliberate practice, were required for this session to succeed. Based on the favorable reviews, the content, case-based format, and directed facilitator feedback essentially remained the same even after the introduction of the manikin in 2016.

We introduced the auscultation manikin to address problems with the delivery of the session. In 2014 and 2015, the students commented on the difficulty of not having the surface anatomy to refer to while auscultating and on the poor sound quality of the online program. Those issues were addressed with the introduction of the manikin in the third year of the course (2016). After the implementation of the manikin, our data showed an upward trend in ratings from 2016 to 2018, with a slight decrease in 2019. The average rating in 2019 was still well above the rating achieved from the initial launch of these sessions.

In analyzing the student comments, it is apparent that strong faculty development is necessary to deliver this session optimally. The year following the introduction of the manikin, the facilitators did not appear confident with the new technology. As faculty became more familiar with the technology, the evaluations improved. There was a slight decrease in student satisfaction between 2018 and 2019, which was believed to be due to suboptimal recruitment of faculty. It is ideal to have a cadre of consistent and experienced facilitators to teach this session, so they become accustomed to the technology. Since fewer experienced faculty were available in 2019, we had relied on facilitators who had not previously taught the session. These novice facilitators spent a majority of the time focused on the large-group cases at the expense of direct auscultation of the manikin. Consequently, student evaluations highlighted a sense of disconnect between the faculty's perception of the session structure compared to student expectations. Additionally, class size was increased in 2019, which led to more students per group. This may have decreased the amount of hands-on time students were able to spend with the manikin, causing the downtrend in evaluations of the session. We initially allocated 5 minutes per pair of students to directly auscultate the manikin, but this appeared to not have been enough time for hands-on practice. The larger class size decreased the faculty-to-student ratio, therefore diminishing the opportunity for direct and specific feedback from the facilitator.

Limitations

The students perceived an increase in learning of lung sounds based on their course evaluations. We focused on the students' reaction to the training on lung sounds (Kirkpatrick level I) and were unable to adequately measure if learning occurred (Kirkpatrick level 2).[17] The evaluations, designed by our institution, gauged students' perceptions of the efficacy of the course in achieving the learning objectives. This evaluation design took a more student-centric approach rather than an objective, assessment-based one. The learning objectives of the session were tested in the summative assessment. However, the exam sampled only a few related questions, so not enough quantitative data were available to make a reliable conclusion as to whether our objectives had been met and whether the introduction of the manikin and the increased student satisfaction were associated with improvement of learning.

The student feedback on the lung sound module was obtained from a 5-point Likert scale and open-box comment evaluations that were required by the institution. A survey including specific questions on content, format, and implementation of the module would have provided a more robust evaluation instrument. Even though we were limited by using a standard institutional survey instrument, the open-box comments from students on their perception of achieving the learning objectives and on their suggestions for improvement of the session were consistent when comparing answers on weekly and end-of-unit surveys and across the class.

This session was held once during our cardiopulmonary block, and therefore, opportunities for spaced repetition were limited. Aside from one after-hours practice session with NPEs, students were not given the opportunity to revisit the material at a later date in preparation for the end-of-unit summative exam. Several students expressed the desire for more individual and hands-on practice. Further practice with faculty facilitators would be helpful to reinforce skills learned during the initial session and to give students more one-on-one practice time with the manikins.

Copyright © 2021 Kaminsky et al. This is an open-access publication distributed under the terms of the Creative Commons Attribution license.

While students were given the opportunity to evaluate the session, faculty facilitators were not similarly asked for formal input. Future feedback from facilitators should address the quality and content of faculty development prior to the session, as well as the ease of teaching the session.

Additionally, it was difficult for us to guarantee adequate student preparation for the session because the lecture on lung sounds and prereadings were not mandatory at our institution, and we did not have a presession assessment to assure readiness.

Future Opportunities
Adopters of this module could develop a graded presession assessment to assure students' uniform baseline knowledge. The results of this pretest could help guide deliberate practice opportunities. Furthermore, one could consider developing a posttest to assess immediate retention of the material. It would be beneficial to create a more robust summative assessment utilizing more questions that directly align with the learning objectives. Pre- and posttests should be administered to students in order to evaluate whether they are actually learning while still feeling engaged in the activity.

For effective learning from this module, it is important to allow sufficient time to provide students with opportunities for repetition or refinement of auscultation skills. Many students requested more direct auscultation time with the manikin to practice listening to and interpreting the lung sounds taught in the session. Possible solutions to this challenge could be to hold more sessions, lengthen the duration of the sessions, or increase the number of manikins, which might not be cost-effective at all institutions. Other options include dividing the activity into two separate sessions, although this might require greater faculty availability. NPEs could be utilized to help facilitate subsequent sessions or to hold after-hours review sessions and reinforce what has been previously learned. It would also be helpful to provide students with increased access to the simulation manikins with appropriate supervision. Given the cost of the manikins, it is recommended that someone familiar with operating the equipment be available during individual student review times.

Favorable student evaluations of this session were related to faculty experience and comfort with the technology. Faculty development should be expanded to assure that facilitators are provided with sufficient training prior to the sessions to best ensure student learning. Facilitators should be keenly aware of the timing and structure of the session, as delineated in the facilitator manual. Ensuring that faculty receive orientation to the lab, including learning objectives, session structure, and overall expectations, remains crucial to the student learning experience. Faculty development could be improved by giving facilitators more time to practice operating the manikins and running through the cases prior to the session. This would ensure that all facilitators have a similar level of familiarity and comfort with the expectations and equipment beforehand.

Appendices

A. Programming List.docx

B. Facilitator Manual.docx

C. Student Manual.docx

D. Postlab Discussion.docx

E. Student Feedback Form.docx

All appendices are peer reviewed as integral parts of the Original Publication.

Jennifer Kaminsky, MD: Resident Physician, Department of Emergency Medicine, Staten Island University Hospital; ORCID: https://orcid.org/0000-0002-2195-3711

Riccardo Bianchi, PhD: Associate Professor, Department of Physiology and Pharmacology, State University of New York Downstate Health Sciences University; Associate Dean for Foundations of Medicine, College of Medicine, State University of New York Downstate Health Sciences University; ORCID: https://orcid.org/0000-0002-1656-4455

Shirley Eisner, PhD: Associate Professor and Co-Director of Gross Anatomy, Department of Cell Biology, State University of New York Downstate Health Sciences University

Robin Ovitsh, MD: Associate Clinical Professor, Department of Pediatrics, State University of New York Downstate Health Sciences University; Associate Dean for Clinical Competencies, College of Medicine, State University of New York Downstate Health Sciences University

Ana Maria Lopez: Fourth-Year Medical Student, College of Medicine, State University of New York Downstate Health Sciences University

Leanna Smith: Fourth-Year Medical Student, College of Medicine, State University of New York Downstate Health Sciences University

Nawar Talukder, MD: Resident Physician, Department of Internal Medicine, University of Arizona College of Medicine - Phoenix

Antonia Quinn, DO: Associate Clinical Professor, Department of Emergency Medicine, State University of New York Downstate Health Sciences University; Associate Director of Clinical Competencies, College of Medicine, State University of New York Downstate Health Sciences University College of Medicine; ORCID: https://orcid.org/0000-0001-8155-5814

Copyright © 2021 Kaminsky et al. This is an open-access publication distributed under the terms of the Creative Commons Attribution license.

Disclosures
None to report.

Funding/Support
None to report.

Ethical Approval
Reported as not applicable.

References

1. Hafke-Dys H, Bręborowicz A, Kleka P, Kociński J, Biniakowski A. The accuracy of lung auscultation in the practice of physicians and medical students. *PLoS One*. 2019;14(8):e0220606. https://doi.org/10.1371/journal.pone.0220606
2. Bintley HL, Bell A, Ashworth R. Remember to breathe: teaching respiratory physiology in a clinical context using simulation. *Adv Physiol Educ*. 2019;43(1):76-81. https://doi.org/10.1152/advan.00148.2018
3. Ward JJ, Wattier BA. Technology for enhancing chest auscultation in clinical simulation. *Respir Care*. 2011;56(6):834-845. https://doi.org/10.4187/respcare.01072
4. Quinn A, Kaminsky J, Adler A, Eisner S, Ovitsh R. Cardiac auscultation lab using a heart sounds auscultation simulation manikin. *MedEdPORTAL*. 2019;15:10839. https://doi.org/10.15766/mep_2374-8265.10839
5. Gottlieb M, Chan TM, Krzyzaniak SM, Grossman C, Robinson DW, Papanagnou D, eds. *Education Theory Made Practical, Volume 2*. e-Book. Academic Life in Emergency Medicine; 2018.
6. McGaghie WC, Kristopaitis T. Deliberate practice and mastery learning: origins of expert medical performance. In: Cleland J, Durning SJ, eds. *Researching Medical Education*. Wiley Online Library; 2015:219-230. https://doi.org/10.1002/9781118838983.ch19
7. McGaghie WC, Issenberg SB, Cohen ER, Barsuk JH, Wayne DB. Does simulation-based medical education with deliberate practice yield better results than traditional clinical education? A meta-analytic comparative review of the evidence. *Acad Med.* 2011;86(6):706-711. https://doi.org/10.1097/acm.0b013e318217e119
8. Issenberg SB, McGaghie WC, Gordon DL, et al. Effectiveness of a cardiology review course for internal medicine residents using simulation technology and deliberate practice. *Teach Learn Med*. 2002;14(4):223-228. https://doi.org/10.1207/s15328015tlm1404_4
9. Ericsson KA. Deliberate practice and acquisition of expert performance: a general overview. *Acad Emerg Med*. 2008;15(11):988-994. https://doi.org/10.1111/j.1553-2712.2008.00227.x
10. Yudkowsky R, Otaki J, Lowenstein T, Riddle J, Nishigori H, Bordage G. A hypothesis-driven physical examination learning and assessment procedure for medical students: initial validity evidence. *Med Educ*. 2009;43(8):729-740. https://doi.org/10.1111/j.1365-2923.2009.03379.x
11. Allen S, Olson A, Menk J, Nixon J. Hypothesis-driven physical examination curriculum. *Clin Teach.* 2017;14(6):417-422. https://doi.org/10.1111/tct.12581
12. Kraman S. Lung sounds: an introduction to the interpretation of auscultatory findings. *MedEdPORTAL*. 2007;3:129. https://doi.org/10.15766/mep_2374-8265.129
13. Cugell D, Gavriely N, Zellner D. Lung sounds and the stethoscope—a lung sounds primer. *MedEdPORTAL*. 2012;8:9066. https://doi.org/10.15766/mep_2374-8265.9066
14. Field M, Burke JM, McAllister D, Lloyd DM. Peer-assisted learning: a novel approach to clinical skills learning for medical students. *Med Educ.* 2007;41(4):411-418. https://doi.org/10.1111/j.1365-2929.2007.02713.x
15. Bickley LS, Szilagyi PG. *Bates' Guide to Physical Examination and History Taking.* 11th ed. Lippincott Williams & Wilkins; 2012:293-321.
16. Gordon MS, Ewy GA, Deleon AC Jr, et al. "Harvey," the cardiology patient simulator: pilot studies on teaching effectiveness. *Am J Cardiol.* 1980;45(4):791-796. https://doi.org/10.1016/0002-9149(80)90123-x
17. Kirkpatrick D. Great ideas revisited: techniques for evaluating training programs—revisiting Kirkpatrick's four-level model. *Train Dev*. 1996;50(1):54-59.

Received: July 1, 2020
Accepted: November 24, 2020
Published: March 2, 2021

Copyright © 2021 Kaminsky et al. This is an open-access publication distributed under the terms of the Creative Commons Attribution license.
